# The 6 month interim analysis of a randomized controlled trial assessing the quality of life in patients with breast cancer related lymphedema undergoing lymphaticovenous anastomosis vs. conservative therapy

**DOI:** 10.1038/s41598-024-52489-3

**Published:** 2024-01-26

**Authors:** Y. M. J. Jonis, J. A. G. N. Wolfs, S. Hummelink, H. J. P. Tielemans, X. H. A. Keuter, S. van Kuijk, D. J. O. Ulrich, R. R. W. J. van der Hulst, S. S. Qiu

**Affiliations:** 1 Department of Plastic, Reconstructive, and Hand Surgery, Maastricht University Medical Center, 6229HX Maastricht, The Netherlands; 2 Department of Plastic and Reconstructive Surgery, Radboud University Medical Center, 6500 HB Nijmegen, The Netherlands; 3 Department of Clinical Epidemiology and Medical Technology Assessment, Maastricht University Medical Center, Maastricht, The Netherlands

**Keywords:** Breast cancer, Randomized controlled trials, Outcomes research

## Abstract

Breast cancer related lymphedema (BCRL) is a chronic condition with a detrimental impact on psychosocial and physical well-being. Lymphaticovenous anastomosis has shown promising results in alleviating physical symptoms and increasing quality of life in patients with BCRL. The aim of the study is to evaluate the effect on health related quality of life (HrQol) after LVA surgery versus conservative treatment in patients with BCRL. The study is a prospective, multicenter randomized controlled trial. Adult women with unilateral BCRL, with early stage lymphedema and viable lymphatic vessels were included. The primary outcome measure was HrQol measured by the lymphedema functioning disability and health (Lymph-ICF) questionnaire. The secondary outcomes were volume difference measured by the water displacement method; the Upper Extremity Lymphedema (UEL) index; and daily use of the compression garments after 3 and 6 months. For this interim analysis 46 patients per group were included. There was a significant improvement in the domains in physical and mental function in the Lymph-ICF questionnaire in the LVA group after 6 months, (− 16.46 ± 18.5, p < 0.05, − 10.12 ± 29.5, p < 0.05 respectively). However, there was no statistical difference in the total score of the Lymph-ICF after 6 months in both groups (LVA-group; − 8.57 ± 22.6, p > 0.05, CDT-group; − 2.65 ± 18.2, p < 0.05). Furthermore, there was no significant volume reduction in both groups (LVA-group: 20.04 ± 196.40, p = 0.497, CDT: 33.98 ± 189.87, p = 0.236). In the LVA group, 41% partially of completely stopped wearing the compression garments after six months whereas in the CDT group 0% discontinued to use of compression garments. LVA resulted in improvement of the domains physical and mental function of the Lymph-ICF. Limb volume did not significantly improve after 6 months. However, around 42% could completely or partially stopped with the use of compression garments in the LVA group. The current results are promising, however longer follow up is required to assess long term effect of LVA for secondary lymphedema.

**Clinical Trial Registration**: NCT02790021 registered on 03/06/2016

## Introduction

Breast cancer related lymphedema (BCRL) is a chronic condition, characterized by an aberrant accumulation of lymph fluid due to dysfunction of the lymphatic system^1–3^. It is a well-known consequence of breast cancer treatment and it represents an important survivorship topic in patients after breast cancer^4–7^.

BCRL affects approximately 29.4% of breast cancer survivors within 2 years after surgery^1,7^. Axillary lymph node dissection, radiotherapy, mastectomy, number of positive lymph nodes and body mass are all independent risk factors for the development of BCRL^6,8,9^.

Nowadays, Health related quality of life (HrQol) is one of the most relevant outcomes after cancer treatment^10,11^. BCRL is known to have a significant negative impact on physical, psychological, and social well-being^5^. Physical morbidities include skin infections, altered sensation, pain, decreased range of motion, strength, and function. In addition, a larger arm sizes requires women to alter daily activities, clothing, sleeping, employment, and sport. Symptoms of anxiety, depression, sexual dysfunction, disturbance of body image, and social avoidance are often associated with BCRL^12–14^.

Currently, complex decongestive therapy (CDT) is the gold standard for the treatment of lymphedema, consisting of the use of compression garments and manual lymphatic drainage^5,15^. These treatment modalities however are not curative and require lifelong costly maintenance^16^.

The first lymphaticovenous anastomosis (LVA) was described in the 1960 as a novel approach to divert lymphatic fluid through formation of an anastomosis from the lymphatic vessels to adjacent venules^17,18^. The concept was revolutionized by Koshima et al. in the 90 s who introduced the concept of supermicrosurgery in lymphatic surgery^19^. Since then the technique has been refined with the introduction of the microscope and designated supermicrosurgical instruments. LVA surgery has been widely implemented as a surgical treatment of lymphedema and it has shown promising results^20–22^. The aim of this 6-month interim analysis is to show the first results of the first randomized controlled trial (RCT) evaluating HrQol in patients with BCRL undergoing LVA surgery in comparison to conservative treatment.

## Methods

From 2018 to 2022, 100 women with BCRL, with stage 1 or 2a, according to the international society of lymphology (ISL) with viable lymphatic vessels and ICG stage II–III according to Narushima measured by near infrared fluoroscopy (NIRF) were included^23,24^. The patients were allocated into two groups, an LVA group and CDT group. The full protocol for the trial has been previously published^25^. The protocol and related documents were approved by the Dutch Medical ethical assessment committee (NL67059.068.18/ METC18-024) registered on 19/12/2018. Clinical Trial Registration: NCT02790021 registered on 03/06/2016. This study followed the Consolidated Standards of Reporting Trials (CONSORT) reporting guidelines.

### Design

The trial was an multi-center randomized controlled trial (RCT). Patients were included in the Maastricht University Medical Center + , Radboud University Medical Center, Zuyderland Medical Centre and the Canisius-Wilhelmina Hospital in the Netherlands. All eligible patients were invited to participate in the RCT. Written informed consent was obtained from all participants.

### Randomization

Surgeon investigators (S.S.Q, H.T, D.U) determined whether the patients were eligible for surgery. The collection of outcomes was performed at the outpatient clinic (J.W, S.H and Y.J). After inclusion and informed consent, participants were randomly assigned to either the LVA or conservative (CDT) group with a 1:1 allocation as per a computer-generated randomization schedule stratified by site using block randomization. This computer-generated randomization was done within the electronic Case Report Form in CASTOR EDC.

### Interventions

#### CDT

All patients in both groups underwent at least 3 months of CDT prior to inclusion. The patients allocated in the CDT group continued to be treated according to standard protocol known as the ‘Verdonkmethod’ which is implemented in all patients receiving CDT in the Netherlands^25^. The CDT consisted of two stages, the first phase entailed skincare, MLD, exercises aimed at improvement of mobility/range of motion, and compression therapy. The second phase was focused on maintenance of the achieved limb volume reduction through compression therapy with therapeutic elastic compression garment for the arm. Skincare, mobility exercises and MLD is continued in this phase if needed.

Using this standardized protocol we were able to compare the outcomes within the CDT group. The conservative treatment and the frequency is controlled by the skin therapist. During follow up all changes in conservative therapy were noted by each patient in the patient diary.

#### LVA

The eligibility for participation in the study was determined with near infrared fluorescence imaging (NIRF) during the outpatient clinic. 0.01 to 0.04 ml of indocyanine green (ICG) (5 mg/ml) was injected into the second and fourth finger webspace intradermally of the lymphedematous limb. Using NIRF the viable lymph vessels were identified, marked, and photographed^23^.

The procedure was performed under general or local anesthesia. This was left at the surgeon’s discretion. During surgery viable lymphatic collecting vessel and similarly sized adjacent recipient venules were identified in the subdermal plane. Subsequently an anastomosis was performed in an end-to-end fashion with 11–0 suture between the lymphatic collecting vessel and the venule, with the use of a surgical microscope. After the anastomosis was made, the patency was confirmed under the microscope. Between 1 and 5 anastomosis were performed in a lymphedematous arm. The superficial wound was closed using 4–0 Ethilon covered by adhesive plasters.

Two weeks after the surgery the patients were able to continue in the maintenance phase of the CDT protocol.

### Outcomes variables

All data was collected according to a standardized protocol. The primary outcome was HrQoL, which was measured by the lymphedema international classification of functioning (Lymph-ICF) questionnaire (Dutch version)^26^. The Lymph-ICF is a validated quality of life questionnaire for patients with upper extremity lymphedema. The questionnaire is divided into five domains: physical function, mental function, household, mobility, life and social activities. A low score on the questionnaire indicates a better quality of life and a higher score indicates of a bad quality of life. A decrease in the sum of scores of more than 10 points, and an increase of more than 9, was considered statistically significant (p < 0.05). A decrease in the sum of scores of more than 15 points was considered clinically significant^27^.

The secondary outcomes were volume reduction, measured by the water displacement method, circumference reduction measured by the upper extremity lymphedema (UEL) index, the complete or partial discontinuation rate of the use of compression garments, reported through a patient diary. Lastly adverse-, and serious adverse events were reported during follow up^28,29^. The data was collected after 3, 6, 12, 18 and 24 month follow up. The data used for the current article are based on the first six months follow up.

### Statistical analysis

We made the following assumptions for the calculation of the sample size to show a statistically significant and clinically relevant difference in quality of life between treatment groups follow-up as measured with the Lymph-ICF questionnaire:

Comparing LVA group to CDT group, the minimal difference in HRQoL that is considered as clinically relevant is 15 points on the Lymph-ICF questionnaire.

To be able to achieve a power of 80%, a total of 45 patients are needed per treatment group, when the SD is 25%, using an alpha of 0.05. If a drop-out rate (loss-to-follow-up and patients with missing data) of 25% is taken into account, a sample size of 60 patients per study group is required and a total of 120 patients will be randomized. Due to the COVID pandemic outpatient clinic at hospitals in the Netherlands were cancelled and there was a lower inclusion rate. In concordance with the ethical committee an amendment was approved to reduce the sample size to 100 patients considering the low dropout rate (n = 1).

Continuous variables were reported as mean with standard deviation. Categorical data were reported as frequencies. To examine the effect of LVA, the paired-samples t-test was used to evaluate the changes between baseline, 3 and 6 months within the groups for the Lymph ICF questionnaire, the relative volume difference measured by water displacement and the UEL index. To measure the effect of the LVA between groups the independent-samples t-test was performed for the above-mentioned variables.

Furthermore, a linear regression was used to determine the relationship between the number of LVAs, follow-up months, lymphedema onset, BMI and ICG stage and for the HrQoL and volume reduction (measured by volume displacement). Results were expressed as regression coefficient with a 95% confidence interval (CI). The use of compression garments, adverse and serious adverse events were reported as frequency. All analysis were performed with IBM SPSS version 25 (IBM Corp., Armonk, N.Y).

### Ethical approval

The study was carried out in accordance to the declaration of Helsinki. The study was approved by the Ethics Committee of Maastricht University Medical Center on 19 December 2018 (NL67059.068.18/ METC18-024). Written consent has been obtained from all participants.

## Results

One hundred consecutive patients with unilateral BCRL were eligible for inclusion. Ninety-two patients finished at least 6 months follow up and were included in the interim analysis. The other eight patients had not finished the 6 month follow up. The patient characteristics are listed in Table 1. Ninety patients had stage 2a lymphedema according to the ISL classification. Eighty-five patients had an ICG stage III lymphedema. After 6 months one patient in the CDT group had discontinued follow up.

**Table 1:** Demographic and baseline clinical characteristics.

	LVA group	CDT group
Number of patients	46	46
BMI (kg/m^2^) ± SD	26 ± 3.55	27 ± 5.11
Lymphedema onset (months)* ± SD	78 ± 69	81 ± 67
Affected arm (no., %)		
Left arm	26 (56)	27 (58)
Right arm	20 (44)	19 (41)
ISL stage (no.)	I: 0	I: 2
	II: 46	II: 44
ICG stage (no., %)	I: 0	I: 0
	II: 5 (11)	II: 11 (23)
	III: 40 (87)	III: 35 (77)
Smoking (no., %)	2 (4)	1 (2)
Erysipelas preoperative (no., %)	13 (27)	17 (36)
Breast cancer treatment		
Radiotherapy (no., %)	41 (90)	42 (89)
Radiotherapy in the armpit (no., %)	24 (52.2)	21 (44.7)
Sentinel node procedure (no., %)	40 (89)	40 (89)
Axillary lymph node dissection (no., %)	43 (95)	41 (89)
Chemotherapy (no., %)	42 (91)	44 (96)
Hormone therapy (no., %)	33 (72)	32 (69)
Complications (no., %)	14 (30)	11 (24)
LVA number of anastomoses		
1–2 anastomosis	26	
3–4 anastomosis	19	
5 anastomosis	1	
Water displacement		
Inter limb volume difference (mL)	381.76 ± 227.08	391.59 ± 320.20
UEL index		
Affected arm	118.61 ± 14.13	120.52 ± 17.54
Unaffected arm	103.62 ± 10.90	103.62 ± 9.57
Lymph-ICF		
Physical	46.46 ± 20.83	44.93 ± 24.72
Mental	34.08 ± 26.14	30.59 ± 28.38
Household	44.67 ± 25.71	56.20 ± 26.15
Mobility	42.96 ± 22.87	50.54 ± 20.80
Social	40.74 ± 23.09	48.37 ± 25.69
Total	41.99 ± 17.06	45.75 ± 18.89

*Lymphedema onset: duration of lymphedema since first diagnosis.

### Primary outcome

#### HrQoL

In the LVA group the mean difference in the total score of the Lymph ICF between baseline and follow up after three and six months was 8.93 ± 22.71 (p > 0.05) and 8.57 ± 22.56 (p > 0.05), respectively. For the domains physical function and mental function a decrease of more than 10 points was observed after 3 and 6 months, representing a statistically significant improvement (p < 0.05). In the CDT group there was no difference observed in the total score of the Lymph-ICF between baseline and follow up. The mean difference in the total score of the Lymph-ICF was 4.57 ± 14.46 (p > 0.05) after 3 months and 2.65 ± 18.21 (p > 0.05) after 6 months. All data respecting HrQol is presented in Table 2.

**Table 2:** The mean difference in Lymph-ICF score between baseline, 3 and 6 months.

LVA group	0–3 months		0–6 months	
Lymph-ICF	Mean difference ± SD	95% CI	Mean difference ± SD	95% CI
Physical	− 15.24 ± 20.5*^a^	[− 9.16, − 21.33]	− 16.46 ± 18.5*^a^	[− 22.09, − 10.38]
Mental	− 10.10 ± 28.8*	[− 18.65, − 1.56]	− 10.12 ± 29.5*	[− 19.42, − 0.82]
Household	− 4.31 ± 32.4	[− 14.18, 5.54]	− 5.91 ± 32.1	[− 16.05, 4.22]
Mobility	− 5.19 ± 32.2	[− 14.76, 4.38]	− 5.42 ± 30.0	[− 14.88, 4.02]
Social	− 9.21 ± 43.3	[− 18.84, 0.74]	− 5.48 ± 37.2	[− 17.21, 6.24]
Total	− 8.93 ± 22.7	[− 15.68, 2.18]	− 8.57 ± 22.6	[− 15.69, 1.45]
CDT group				
Physical	− 4.93 ± 14.9	[− 9.45, 0.41]	− 0,56 ± 19,1	[− 6.45, 5.31]
Mental	− 3.06 ± 16.2	[− 7.99, 1,85]	− 5.52 ± 22.3	[− 12.38, 1.34]
Household	− 9.15 ± 30.1	[− 17.89, 0.75]	− 7.08 ± 32.0	[− 16.88, 2.77]
Mobility	− 2.27 ± 27.3	[− 10.58, 6.03]	− 2.47 ± 27.3	[− 10.66, 5.71]
Social	2.86 ± 33.0	[− 12.99, 7.28]	3.89 ± 33.3	[− 14.15, 6.36]
Total	− 4.57 ± 14.5	[− 8.98, 0.18]	− 2.65 ± 18.2	[− 8.26, 2.95]

The mean difference in the score of the Lymph-ICF is calculated using the Paired Samples *T*-Test. Values with * indicate a statistically significant difference (p < 0.05).

^a^Indicates clinical significant difference. A negative value indicates an improvement in quality of life.

When comparing the total score of the Lymph-ICF between the two groups a statistical difference was observed in physical function after 3 (p = 0.006) and 6 months (p = 0.001), presented in Table 3. No relationship between the total score of the Lymph-ICF and preoperative ICG stage, the amount of anastomosis, lymphedema onset, and BMI was found (see Table 4).

**Table 3:** The mean difference between groups after 3 and 6 months.

CDT vs. LVA	0–3 months			0–6 months		
	Mean difference	95% CI	P-value	Mean difference	95% CI	P-value
Physical	− 10.69*	[− 18.22, − 3.15]	0.006	− 13.77*	[− 21.84,5.69]	0.001
Mental	− 7.26	[− 17.04, 2.95]	0.146	− 4.06	[− 15.31, 7.19]	0.475
Household	4.64	[− 8.70, 17.98]	0.491	− 0.62	[− 14.71, 13.46]	0.930
Mobility	− 0.33	[− 12.18, 11.52]	0.956	− 4.07	[− 16.09, 7.93]	0.502
Social	− 3.92	[− 6.05, 8.10]	0.518	− 2.93	[− 17.49, 11.62]	0.690
Total	− 3.87	[− 11.74, 4.00]	0.332	− 5.92	[− 14.88, 3.04]	0.192

The mean difference between groups is calculated using the Independent Samples *T*-Test. Values with * indicate a statistically significant difference (p > 0.05).

**Table 4:** Linear regression analysis: using the difference in the Lymph-ICF score as dependent variable.

Independent variable	Difference in Lymph-ICF score^a^		
	B	95% CI	P
Constant	30.57	[− 148.9, 210.0]	0.732
Anastomosis (count)	− 13.12	[− 27.96, 1.69]	0.081
ICG stage	− 11.75	[− 57.45, 33.96]	0.606
Onset lymphedema	− 0.107	[− 0.317, 0.103]	0.309
BMI	2.39	[− 1.73, 6.53]	0.248

Unstandardized beta (B): Calculated using linear regression analysis. A negative value means a decrease in Lymph-ICF, representing an increase in Quality of Life.

^a^Lymph-ICF score difference is calculated by subtracting the post-OR lymph-ICF score from the pre-OR lymph-ICF score.

### Secondary outcomes

#### Volume reduction

The excess volume was measured by the difference in affected and non-affected arm. The absolute volume difference was 24.80 ± 179.93 mL (p = 0.398) after 3 months and 20.04 ± 196.40 mL (p = 0.497) after 6 months for the LVA group. For the CDT group, 13.88 ± 193.36 mL (p = 0.640) and 33.98 ± 189.87 mL (p = 0.236) after three months and six months, respectively. All data respecting volume measurements is presented in Table 5. There was no significant difference observed between the two groups, − 2.82 (p = 0.737) after 6 months.

**Table 5:** The relative difference in volume measured by the WDM after 0–3 and 0–6 months.

	0–3 months			0–6 months		
	WDM					
	Mean absolute difference ± SD	95% CI	P-value	Mean absolute difference ± SD	95% CI	P-value
LVA	24.80 ± 179.93	[− 32.74, 84.34]	0.389	20.04 ± 196.40	[− 38.96, 79.05]	0.497
CDT	13.88 ± 193.36	[− 45.6, 73.39]	0.640	33.98 ± 189.87	[− 23.06, 91.02]	0.236

The relative difference in volume after 0–3 and 0–6 months is calculated using the Paired Samples T-Test.

Furthermore, there was no correlation between volume difference and preoperative ICG stage, the amount of anastomosis, lymphedema onset, and BMI (see Table 6).

**Table 6:** Linear regression analysis: using the difference in volume as dependent variable.

Independent variable	Difference in volume^a^		
	B	95% CI	P
Constant	− 346.301	− 1139.4–455.56	0.373
Anastomosis (count)	42.29	− 34.98–96.89	0.204
ICG stage	110.7	− 62.93–344.33	0.253
Onset lymphedema	0.002	− 1.24–0.65	0.131
BMI	− 3.926	− 23.12–13.57	0.657

Unstandardized beta (B): Calculated using linear regression analysis.

^a^The difference in volume measured by WDM and is calculated by subtracting the post-OR volume from the pre-OR volume.

### Limb circumference

The mean absolute difference in UEL index for the LVA group 3.65 ± 7.24 (p = 0.002) after 3 months and 1.84 ± 14.6, (p = 0.497) after 6 months, for the CDT group the mean absolute difference was respectively 3.30 ± 31.57 (p = 0.521) and − 0.84 ± 14.6 (p = 0.189) after three and six months. The data is presented in Table 7*.* Furthermore, there is no significant difference in UEL index between the LVA and CDT group, 2.43 (p = 0.458).

**Table 7:** The relative difference in circumference after 0–3 and 0–6 months.

	0–3 months			0–6 months		
	UEL-index					
	Mean absolute difference ± SD	95% CI	P-value	Mean absolute difference ± SD	95% CI	P-value
LVA	3.65 ± 7.24	[1.42, 5.88]	0.002	1.84 ± 9.14	[− 0.94, 4.62]	0.189
CDT	3.30 ± 32.7	[− 6.93, 13.47]	0.521	− 0.84 ± 14.6	[− 5.17,3.49]	0.189

The relative difference in circumference after 0–3 and 0–6 months is calculated using the Paired Samples *T*-test.

### Discontinuation of compression garments

After 3 months 8 patients (17.0%) in the LVA group completely discontinued the use of compression garments. After 6 months the discontinuation rate increased to 10 patients (21.3%). Furthermore after 3 and 6 months, 10 patients (21.7%) partially discontinued use of compression garments. None of the patients in the CDT group discontinued the use of compression garments in the first 6 months.

### Adverse events (AE)

Within the first 3 months 7 AE’s were observed.

In the CDT group one subject had a mild episode erysipelas, treated with oral antibiotics and in one patient moderate erysipelas occurred, where treatment with intravenous antibiotics were required. One patient had COVID, one patient was diagnosed with muscular rheumatism.

In the LVA group one patient had moderate erysipelas, treated with intravenous antibiotics, one patient had a skin infection and 1 patient had pneumonia.

After 6 months, 5 AEs occurred in the CDT group and 3 AE in the LVA group occurred. In the CDT group 4 patients had erysipelas, in the LVA group 3 patients. All patients received oral antibiotics for the treatment of erysipelas. One patient in the LVA group had an allergic reaction of unknown origin.

### Severe adverse events (SAE)

After 3 months no SAE were reported. After six months one patient in the LVA group reported recurrence of her breast cancer but she remained in the study.

## Discussion

Previous published studies on the efficacy of the LVA surgery have shown promising results^30–33^. A decrease on subjective complaints and a volume reduction between 0 and 61% have been reported^34–36^. However, most of the studies included a small heterogeneous population and have a retrospective design^28,37–40^. The reported studies did not compare LVA surgery with other conservative treatment modalities and use different assessment tools resulting in disparate results^35,41,42^.

To our knowledge, this the first large scale prospective randomized multicenter study assessing the effectiveness of LVA surgery compared with conservative treatment in patients with BCRL with HrQoL as primary outcome. Currently, there is a wide variety of quality of life questionnaires in lymphedema. The Lymph-ICF for the upper extremity has been well rated in regards to content validity, reliability, and construct validity based on good-quality evidence^43^.

Intra group analyses demonstrated that physical function and mental function were significantly improved in the LVA group after 3 and 6 months. Moreover, the physical function was significantly improved in the LVA group compared to the CDT group. Indicating that the patients in the LVA group experience a significant improvement in physical symptoms such as heaviness, swelling, weakness, tingling and tightness of the arm as early as 3 months after LVA surgery. Previous studies reported an overall improvement of the subjective symptoms after LVA surgery^21,37,44,45^. However so far, the total score of the Lymph-ICF in the LVA and CDT group showed no statistically significant improvement.

Notwithstanding, the total score of the Lymph-ICF in the LVA group are promising in comparison to the CDT group where smaller differences in total score of the Lymph-ICF were seen. Furthermore, there was a high variability in the overall cohort. Moreover, patients with lymphedema present a wide array of complaints and symptoms. While some patients experience a detrimental effect in the arm volume, other patients experience more loss of mobility, pain and heaviness^5^.

After 3 and 6 months no significant changes in arm volume were observed for both groups. Interestingly, after 3 months UEL index significantly improved in the LVA group, however this trend did not persist after 6 months. The volume reductive effect of the LVA treatment could not be evidenced during the first 6 months in the current study. This is concordant with previous studies^21,25,45^.

However, the absence of volume reduction does not equate treatment failure. Even though the population had early stage lymphedema a discrepancy was seen between the ISL stage and ICG stage. Most of the cohort had ISL-stage 2a lymphedema, however using NIRF we mostly saw patients with ICG stage 3. Moreover, the patients in our cohort have had lymphedema for an average of ~ 7 years. Altogether this could indicate that even in early stage (ISL stage 2a) lymphedema determined by the ISL classification, the lymph vessels in the subdermal plane could already be nonfunctioning. Furthermore, in ISL stage 2a lymphedema changes in the tissue have been established using Dual Energy X-Ray Absorptiometry, such as lymph vessel dysfunction, fat deposition and, fibrosis. However, little is known about the exact physiological progression of lymphedema over time, which makes it difficult to establish which patients will benefit from LVA surgery^46–49^.

Other systematic reviews have demonstrated that limb circumference significantly decreased, however these studies have a smaller patient population, reported heterogeneous assessment modalities and a had longer follow up^39,50^. There are many factors that might influence the outcome of the LVA, however there is a lack of consensus on what these factors are^42,51–53^. A positive correlation between Lymph-ICF and the amount of anastomosis has been reported^21^. As of yet, in our study no correlation was found between Lymph-ICF the amount of anastomosis, ICG stage, BMI and onset of lymphedema. However, in the study by Qiu et al. patients received multiple LVA surgeries and a longer follow up period then our current cohort^21^.

In our study there was no correlation shown between volume reduction and the number of anastomosis performed. This concurs with the study conducted by Winters et al. where there was no correlation between the amount of anastomosis and volume reductive effect of the LVA^28^. In the current cohort there was no relationship between the volume, the duration and severity of lymphedema. Concurrently, a meta-analysis conducted by Nacchiero et al. reported that the stage and duration of lymphedema were not dependent on the success of the operation^39,54^.

Lastly approximately 42% of the patient population in the LVA group completely of partially discontinued the use of compression garments. The discontinuation rate in our population is line with previously published articles^21,44,45^. Unfortunately, we did not include the reason for discontinuation in our questionnaire, which might be interesting to investigate in the future.

This study is a 6-month interim analysis where 92% of the population completed six-month follow up. At the time of analysis, one patient had dropped out. Because the design of the current study eliminates any form of selection bias, this study more closely resembles results of the general population in comparison to other smaller and retrospective studies. In our study, patients were only able to receive one LVA surgery whereas in other previously published studies patients were able to receive more sessions, and ultimately more LVA’s^21,55^. Although the results of the first 6 months may be encouraging, a longer follow up period is required the assess the true effect of the LVA in comparison to CDT for secondary lymphedema.

## Conclusion

Lymphaticovenous anastomosis resulted in improvement of physical and mental function in patients with BCRL. Limb volume and limb circumference did not significantly improve after 6 months in both groups. However, in the LVA group around 40% could completely or partially stopped with the use of compression garments. The current results are promising, however longer follow up is required to assess the long term effect of LVA.

## Data Availability

The datasets used and/or analysed during the current study are available from the corresponding author on reasonable request.

## Abbreviations

AE: Adverse events
BCRL: Breast cancer related lymphedema
BMI: Body mass index
CDT: Complex decongestive therapy
CI: Confidence interval
HrQoL: Health related quality of life
ICG: Indocyanine green
ISL: International society of lymphology
LVA: Lymphaticovenous anastomosis
Lymph-ICF: Lymphedema functioning, disability and health questionnaire
MLD: Manual lymphatic drainage
NIRF: Near infrared fluoroscopy
RCT: Randomized controlled trial
SAE: Severe adverse events
UEL-index: Upper extremity lymphedema index
VAS: Visual analogue score
WDM: Water displacement method

## References

[1] Gillespie TC (2018). Breast cancer-related lymphedema: Risk factors, precautionary measures, and treatments. Gland Surg..

[2] Fu MR (2014). Breast cancer-related lymphedema: Symptoms, diagnosis, risk reduction, and management. World J. Clin. Oncol..

[3] Wanchai A (2016). Breast cancer-related lymphedema: A literature review for clinical practice. Int. J. Nurs. Sci..

[4] Morrell RM (2005). Breast cancer-related lymphedema. Mayo Clin. Proc..

[5] Anbari AB, Wanchai A, Armer JM (2021). Breast cancer-related lymphedema and quality of life: A qualitative analysis over years of survivorship. Chronic Illn..

[6] Petrek JA, Heelan MC (1998). Incidence of breast carcinoma-related lymphedema. Cancer.

[7] Hayes SC (2008). Lymphedema after breast cancer: Incidence, risk factors, and effect on upper body function. J. Clin. Oncol..

[8] Kwan ML (2010). Risk factors for lymphedema in a prospective breast cancer survivorship study: The Pathways Study. Arch. Surg..

[9] Ren Y (2022). Burden of lymphedema in long-term breast cancer survivors by race and age. Cancer.

[10] Kim K, Yoon H (2021). Health-related quality of life among cancer survivors depending on the occupational status. Int. J. Environ. Res. Public Health.

[11] de Ligt KM (2019). The impact of health symptoms on health-related quality of life in early-stage breast cancer survivors. Breast Cancer Res. Treat..

[12] Ahmed RL (2008). Lymphedema and quality of life in breast cancer survivors: The Iowa Women's Health Study. J. Clin. Oncol..

[13] Pusic AL (2013). Quality of life among breast cancer patients with lymphedema: A systematic review of patient-reported outcome instruments and outcomes. J. Cancer Surviv..

[14] Rockson SG (2019). Cancer-associated secondary lymphoedema. Nat. Rev. Dis. Primers.

[15] Zasadzka E (2018). Comparison of the effectiveness of complex decongestive therapy and compression bandaging as a method of treatment of lymphedema in the elderly. Clin. Interv. Aging.

[16] Ridner SH (2005). Quality of life and a symptom cluster associated with breast cancer treatment-related lymphedema. Support Care Cancer.

[17] Nielubowicz J, Olszewski W (1968). Surgical lymphaticovenous shunts in patients with secondary lymphoedema. Br. J. Surg..

[18] Jacobson JH, Suarez EL (1962). Microvascular surgery. Dis. Chest.

[19] Koshima I (2000). Supermicrosurgical lymphaticovenular anastomosis for the treatment of lymphedema in the upper extremities. J. Reconstr. Microsurg..

[20] Park KE (2020). Surgical management of lymphedema: A review of current literature. Gland Surg..

[21] Qiu SS (2020). Outcomes following lymphaticovenous anastomosis (LVA) for 100 cases of lymphedema: Results over 24-months follow-up. Breast Cancer Res. Treat..

[22] Chang DW, Suami H, Skoracki R (2013). A prospective analysis of 100 consecutive lymphovenous bypass cases for treatment of extremity lymphedema. Plast. Reconstr. Surg..

[23] Narushima M (2016). Indocyanine green lymphography findings in limb lymphedema. J. Reconstr. Microsurg..

[24] The Diagnosis and Treatment of Peripheral Lymphedema (2016). 2016 consensus document of the international society of lymphology. Lymphology.

[25] Wolfs J (2020). Improving the quality of life of patients with breast cancer-related lymphoedema by lymphaticovenous anastomosis (LVA): Study protocol of a multicentre randomised controlled trial. BMJ Open.

[26] Devoogdt N (2011). Lymphoedema functioning, disability and health questionnaire (Lymph-ICF): Reliability and validity. Phys. Ther..

[27] De Vrieze T (2019). Revision of the lymphedema functioning, disability and health questionnaire for upper limb lymphedema (Lymph-ICF-UL): Reliability and validity. Lymphat. Res. Biol..

[28] Yamamoto T (2013). Upper extremity lymphedema index: A simple method for severity evaluation of upper extremity lymphedema. Ann. Plast. Surg..

[29] Sagen A (2009). Validity for the simplified water displacement instrument to measure arm lymphedema as a result of breast cancer surgery. Arch. Phys. Med. Rehabil..

[30] Mihara M (2014). Lymphaticovenular anastomosis to prevent cellulitis associated with lymphoedema. Br. J. Surg..

[31] Auba C (2012). Lymphaticovenular anastomoses for lymphedema treatment: 18 months postoperative outcomes. Microsurgery.

[32] Akita S (2014). Suitable therapy options for sub-clinical and early-stage lymphoedema patients. J. Plast. Reconstr. Aesthet. Surg..

[33] Chen WF (2016). Indocyanine green lymphographic evidence of surgical efficacy following microsurgical and supermicrosurgical lymphedema reconstructions. J. Reconstr. Microsurg..

[34] Baumeister RG, Siuda S (1990). Treatment of lymphedemas by microsurgical lymphatic grafting: What is proved?. Plast. Reconstr. Surg..

[35] Carl HM (2017). Systematic review of the surgical treatment of extremity lymphedema. J. Reconstr. Microsurg..

[36] Masià J, Pons G, Rodríguez-Bauzà E (2016). Barcelona lymphedema algorithm for surgical treatment in breast cancer-related lymphedema. J. Reconstr. Microsurg..

[37] Winters H (2017). The efficacy of lymphaticovenular anastomosis in breast cancer-related lymphedema. Breast Cancer Res. Treat..

[38] Yamamoto T (2013). Navigation lymphatic supermicrosurgery for the treatment of cancer-related peripheral lymphedema. Vasc. Endovasc. Surg..

[39] Nacchiero E (2020). Lymphovenous anastomosis for the treatment of lymphedema: A systematic review of the literature and meta-analysis. Lymphology.

[40] Will PA (2022). Supermicrosurgical treatment for lymphedema: A systematic review and network meta-analysis protocol. Syst. Rev..

[41] Wang S (2021). Supermicrosurgical lymphoevenous anastomosis for the treatment of peripheral lymphedema: A systematic review of the literature. Chin. J. Plast. Reconstr. Surg..

[42] Rosian K, Stanak M (2019). Efficacy and safety assessment of lymphovenous anastomosis in patients with primary and secondary lymphoedema: A systematic review of prospective evidence. Microsurgery.

[43] Paramanandam VS (2021). Self-reported questionnaires for lymphoedema: A systematic review of measurement properties using COSMIN framework. Acta Oncol..

[44] Wolfs J (2020). Correlation between patency and clinical improvement after lymphaticovenous anastomosis (LVA) in breast cancer-related lymphedema: 12-month follow-up. Breast Cancer Res. Treat..

[45] Cornelissen AJM (2017). Lymphatico-venous anastomosis as treatment for breast cancer-related lymphedema: A prospective study on quality of life. Breast Cancer Res. Treat..

[46] Mihara M (2018). Lymphatic venous anastomosis can release the lymphedema-associated pain of upper limb after breast cancer treatment. J. Reconstr. Microsurg. Open.

[47] Garza RM (2019). The relationship between clinical and indocyanine green staging in lymphedema. Lymphat. Res. Biol..

[48] Zhang J, Hoffner M, Brorson H (2022). Adipocytes are larger in lymphedematous extremities than in controls. J. Plast. Surg. Hand Surg..

[49] Jørgensen MG (2021). Indocyanine green lymphangiography is superior to clinical staging in breast cancer-related lymphedema. Sci. Rep..

[50] Basta MN, Gao LL, Wu LC (2014). Operative treatment of peripheral lymphedema: A systematic meta-analysis of the efficacy and safety of lymphovenous microsurgery and tissue transplantation. Plast. Reconstr. Surg..

[51] Tsai P-L (2020). Determining factors in relation to lymphovascular characteristics and anastomotic configuration in supermicrosurgical lymphaticovenous anastomosis—A retrospective cohort study. Int. J. Surg..

[52] Klingelhöfer E (2019). Factors affecting outcomes after supermicrosurgical lymphovenous anastomosis in a defined patient population1. Clin. Hemorheol. Microcirc..

[53] Chen WF (2018). How to get started performing supermicrosurgical lymphaticovenular anastomosis to treat lymphedema. Ann. Plast. Surg..

[54] Chang DW (2010). Lymphaticovenular bypass for lymphedema management in breast cancer patients: A prospective study. Plast. Reconstr. Surg..

[55] Pandey SK (2021). Supermicrosurgical lymphaticovenular anastomosis <i>vs. </i> vascularized lymph vessel transplant—Technical optimization and when to perform which. Plast. Aesthet. Res..

